# Comparative Pan-Genomic Analysis Revealed an Improved Multi-Locus Sequence Typing Scheme for *Staphylococcus aureus*

**DOI:** 10.3390/genes13112160

**Published:** 2022-11-19

**Authors:** Maira Jalil, Fatima Quddos, Farha Anwer, Samavi Nasir, Abdur Rahman, Metab Alharbi, Abdulrahman Alshammari, Huda Kamel Alshammari, Amjad Ali

**Affiliations:** 1 Atta Ur Rahman School of Applied Biosciences (ASAB), National University of Sciences and Technology (NUST), Islamabad 44000, Pakistan; 2 Department of Biology, University of Virginia College and Graduate School of Arts & Sciences, Charlottesville, VA 22903, USA; 3 Translational Biology, Medicine, & Health Graduate Program at Virginia Tech, 1 Riverside Circle Suite 201, Roanoke, VA 24016, USA; 4 Department of Pharmacology and Toxicology, College of Pharmacy, King Saud University, P.O. Box 2455, Riyadh 11451, Saudi Arabia; 5 Department of Pharmacy, Riyadh Security Forces Hospital, Ministry of Interior, Riyadh 11564, Saudi Arabia

**Keywords:** multi-locus sequence typing (MLST), *Staphylococcus aureus*, methicillin-resistance *Staphylococcus aureus* (MRSA), pangenome, comparative genome analysis

## Abstract

The growing prevalence of antibiotic-resistant *Staphylococcus aureus* strains mandates selective susceptibility testing and epidemiological investigations. It also draws attention to an efficient typing strategy. Whole genome sequencing helps in genetic comparison, strain differentiation, and typing; however, it is not that cost-effective. In comparison, Multi-Locus Sequence Typing (MLST) is an efficient typing method employed for bacterial strain typing and characterizations. In this paper, a comprehensive pangenome and phylogenetic analysis of 502/1279 *S. aureus* genomes is carried out to understand the species divergence. Additionally, the current Multi-Locus Sequence Typing (MLST) scheme was evaluated, and genes were excluded or substituted by alternative genes based on reported shortcomings, genomic data, and statistical scores calculated. The data generated were helpful in devising a new Multi-Locus Sequence Typing (MLST) scheme for the efficient typing of *S. aureus* strains. The revised scheme is now a blend of previously used genes and new candidate genes. The genes *yQil*, *aroE*, and *gmk* are replaced with better gene candidates, *opuCC*, *aspS*, and *rpiB*, based on their genome localization, representation, and statistical scores. Therefore, the proposed Multi-Locus Sequence Typing (MLST) method offers a greater resolution with 58 sequence types (STs) in comparison to the prior scheme’s 42 STs.

## 1. Introduction

*S. aureus*, both commensal and pathogenic, inhabits humans’ noses, mucosal passages, and skin, which can result in mild to severe infections, sometimes resulting in death [1]. The infections can spread through inhalation, direct contact, and contamination [2]. The statistics of its infections occurring worldwide and its isolation in healthcare settings increase the need for effective typing schemes for close monitoring of various strains and lineages.

According to the Centers for Disease Control’s (CDC) (https://www.cdc.gov/ (accessed on 15 September 2022)) 2019 report “Antibiotic Resistance Threats in the United States, 2019”, antibiotic resistance of methicillin-resistant *S. aureus* is ranked ninth in the list of serious threats [3]. In 2011, according to CDC, the invasive MRSA infection cases were 80,451. Of these cases, approximately 11,285 deaths occurred in the United States alone [4]. *S. aureus* was the second most isolated microbe from bacteremia in Europe in 2008 with increasing prevalence over time. In Wales and England, the statistics report that there has been an overall increase in the deaths caused by *S. aureus*, especially MRSA, increasing from 430 in 1993 to 638 in 2011 [5]. According to a report from the United States, around 39–60% of the samples isolated from clinics of *S. aureus* show methicillin resistance. In comparison to Methicillin-Susceptible *S. aureus* (MSSA) strains, Methicillin-Resistance *S.aureus* (MRSA) strains are related to greater mortality incidents [6]. There is a major risk of the emergence of a super-resistant bug since *S. aureus* is known for its acquisition of antibiotic-resistant genes. There are several types of infections that *S. aureus* is responsible for, ranging from mild infections like skin boils to life-threatening infections like entering the blood circulatory system and infecting the internal vital organs [6]. The commonly reported infections that are reported are not limited to superficial skin infections e.g., boils, sties, formation of abscesses, osteomyelitis, endocarditis, nosocomial infections, food poisoning through enterotoxins, toxic shock syndrome, bacteremia, and sepsis [7]. For this reason, the typing of *S. aureus* isolates is very important. The current typing methods fail to characterize the rapidly evolving *S. aureus* strains.

The labor and high cost remain a challenge for many while adopting whole genome sequencing. Otherwise, in theory, it is an ideal approach for the identification and typing of strains followed by genomic diversity and relatedness in species comparisons [8,9]. On the other hand, the number of completely sequenced genomes of *S. aureus* is increasing day by day in publicly available databases. Consequently, new methods are being developed to analyze and compare these genomic sequences in a faster and more cost-effective manner. Pangenome comparative analysis is one such method. This method utilizes the complete genetic repertoire of all the available strains and gives an overall picture of the evolution, recombination, gene acquisition and geneloss, and recombination in these strains. A particular bacterial species can be efficiently represented by its pan-genome which is comprised of the core genome (genes that are present in all strains of that particular species and are likely to be involved in functions related to the vitality of the cell) and the dispensable genome (genes that are either unique, present in only a single strain or shared by more than two genomes) [10].

With the help of pan-genome analysis, core genes can be isolated. These isolated genes can then be utilized in the shortlisting of genes that can be used in MLST. According to the literature review, MLST is the most efficient typing method for *S. aureus*. MLST is a widely used and quite reliable technique for the typing and sorting of different strains of a species in different groups by using housekeeping genes.

The MLST scheme was initially developed for Neisseria meningitidis in 1998 [11]. Typically, an MLST scheme utilizes around six to eight housekeeping genes. It utilizes an internal fragment of ~600 bp of housekeeping genes in the characterization of lineages of bacteria [12]. MLST genes represent the whole genome and the data from these genes are comparable to the phylogenetic tree based on complete genomes analysis [13]. Hence, instead of analyzing the whole genome, these representative genes can type the isolates and help in understanding evolutionary changes within the microorganism for population studies [14].

There are several reasons for using MLST as a typing method: (a) nucleotide sequence data are the best way to analyze the population as they are unambiguous and generic; (b) the scheme can represent the population structure that is required for many species to represent the congruency of the clones; (c) MLST is easily accessible and can type microbial clones globally; and (d) the data of the scheme are reproducible [15].

The evaluation of MLST schemes is necessary for their efficient working. The increased resistance patterns and the whole genome sequenced data mandates to analyze the scheme with time. According to the literature review, due to variation in the 12 bp, 3′ end overlap region of *gmk* is difficult to amplify in the laboratory, which led to difficulty in the typing of *S. aureus* strains [16]. Moreover, the dN/dS value for *aroE* and *gmk* are 12.60328 and 4.06854, which indicates the instability of the genes. The present MLST scheme represents a portion of the whole genome where approximately 43% of the genomic area remains unrepresented in the present scheme. This shortcoming can result in failure to represent the genome phylogeny. This calls for our attention to the improvement and revision of the current scheme to understand the current emerging patterns of *S. aureus*.

The rapid evolution of the genome of *S. aureus* as indicated by the comparative pan-genome analysis and the increasing evidence of the limitation of the genes of the MLST scheme of *S. aureus* from the literature was the major reason for the assessment of the current scheme [17].

## 2. Materials and Methods

This study was comprised of two main components both of which were linked to each other: comparative pan-genomic analysis, and the development of an improved MLST scheme. The results of the pangenome analysis were used extensively in identifying the pan and core genes for the *S. aureus* genome, possible gene candidates for the MLST scheme, studying the evolution of *S. aureus* on a molecular and functional basis, configuring an improved MLST scheme, analyzing the pathogenicity, estimating the gene acquisition and loss, investigating the recombination in *S. aureus,* and estimating how open the genome of *S. aureus* is.

**Figure 1:**
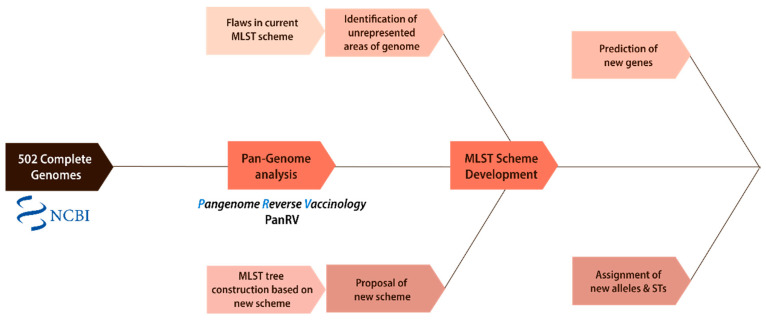
Overall methodology. The flowchart demonstrates the overall methodology followed in this study. It shows the two main components: phylogenetic analysis and the development of a new MLST scheme.

The details of each of the components are discussed in Figure 1 below.

### 2.1. Sequence Retrieval and S. aureus Genome Data and Gene Annotation

**Figure 2:**
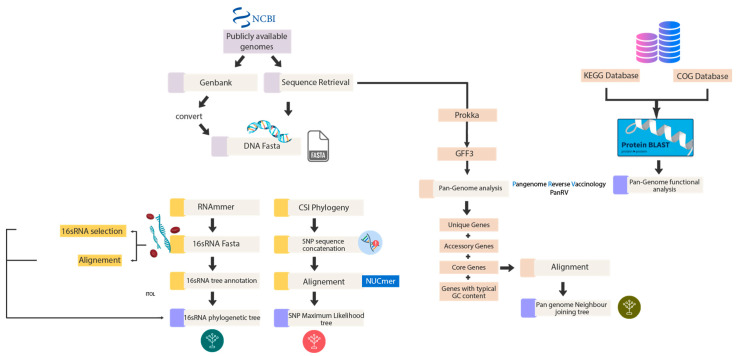
Phylogenetic analysis. The flowchart demonstrates the first component of the methodology, the phylogenetic analysis, outlining the software in detail.

The total genomic sequences available for *S. aureus* to date are 1279, and 502 out of 1279 were downloaded from National Center for Biotechnology Information (NCBI) (https://www.ncbi.nlm.nih.gov/ (accessed on 21 October 2022)) and further subjected to different genomic analysis. DNA sequences in FASTA format were used to make a 16s rRNA phylogenetic tree and an SNP-based phylogenetic tree. DNA sequences in FASTA format were annotated by Prokka at default parameters for estimation of pan-genome [18], a tool for the complete annotation of the bacterial draft genome in approximately 10 min. The steps are further elaborated in Figure 2.

### 2.2. Pan-Genome Profile Analysis

We first performed Pangenome analysis using PanRV in-house pipeline to analyze the 502/1279 complete genome sequences. PanRV is a pangenome reverse vaccinology tool that assesses the genome against each category, such as pan, core, accessory, and unique, using a pangenome estimate module built on roary. The output file from prokka in GFF3 format was needed as input in running the pangenome estimation module (PGM) of a PanRV [19]. The steps followed and the software used are elaborated in Figure 2 above.

### 2.3. Development of an Improved MLST Scheme

For the current scheme, the literature was reviewed to identify the problems of the current MLST scheme. Meanwhile, the data of the current scheme were subjected to further analysis using different software. The allelic profile for 502 genomes was determined using PubMed. The MLST tree of the 502 genomes was constructed, and clones were identified.

**Figure 3:**
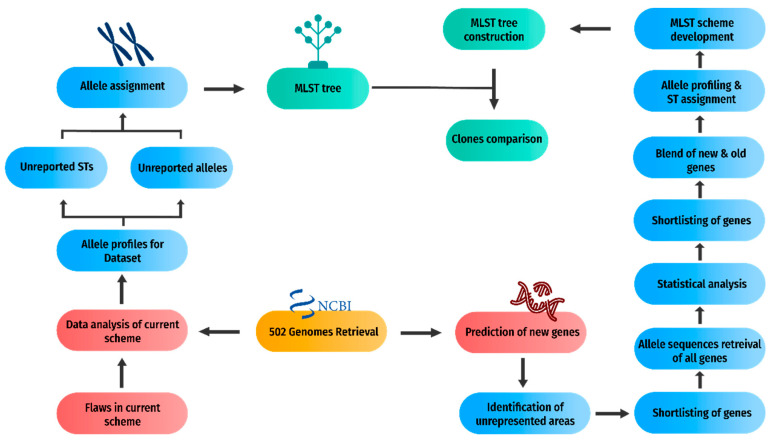
Development of a new MLST scheme. The flowchart demonstrates the second component of the methodology, the development of a new MLST scheme, in detail.

The prediction of new gene candidates for the new MLST scheme was the second part of the methodology depicted in Figure 3 for which an inclusion–exclusion criterion based on the size and unique alleles was established based on the literature review and drawbacks of the current scheme.

Allelic profiles of all the genes were retrieved using PubMed. These proposed gene candidates then underwent statistical analysis that led to further shortlisting of the genes. A new scheme constituting proposed gene candidates and current scheme candidates was constructed. The new scheme eliminated all the genes of the current MLST scheme having lower shortcomings in satisfying the new criteria. Finally, STs were assigned for the proposed MLST scheme. Subsequently, an MLST tree was constructed. This tree was compared with the current MLST tree, and insights were drawn.

#### 2.3.1. Population Structure

The population structure of *S. aureus* developed using the current MLST scheme was compared with that of the newly proposed scheme. For this purpose, the seven loci of housekeeping genes of 502 genomes (available at the time of analysis) were subjected to MLST scheme analysis and were assigned allele numbers based on the already reported MLST scheme [20].

#### 2.3.2. New Gene Candidates for *S. aureus*

The purpose of this scheme is to represent the whole genome and to select genes with a higher discriminatory index than the previously reported genes. The higher the index, the greater the ability to differentiate between strains. Genes that play crucial roles in cellular pathways were selected with the help of BacMap, UniProt, and COG. The following are the criteria for the selection of candidate genes for the MLST scheme.

#### 2.3.3. Core genes

In the selection process, the two parameters of size and core genome presence were kept side by side. Core genome analysis means that a particular gene is present throughout the species [13]. Each gene was searched on PubMed blast Sequence query for its presence in the core genome.

#### 2.3.4. Role in Cellular Pathways

These housekeeping genes are constitutively active and play a crucial role in cellular mechanisms. Genes playing roles in transcription, translation, recombination, repair, defense mechanism, cell cycle regulation, cell division, and chromosome partitioning were selected. Moreover, the core genomes in FASTA selected from the PanRV output were subjected to COG analysis for functional annotation. The sequences of the genes were also retrieved using the online database “BacMap Genome Atlas” (http://bacmap.wishartlab.com/ (accessed on 23 October 2022)), Uniprot (www.uniprot.org/ (accessed on 23 October)) [21,22,23,24].

#### 2.3.5. Mobile Elements

*S. aureus* is commonly known for recombination events within the core genome, which could be attributed to the presence of mobile elements [25]. Therefore, each candidate gene was looked for the presence of mobile elements in or around the proximity of candidate genes through an online tool, CENSOR by “Giri Repbase” (http://www.girinst.org/censor/index.php (accessed on 22 October 2022)) [26]. Then 100 bp upstream and downstream of each gene was looked up for the mobile elements using the same software.

#### 2.3.6. Size of the Gene

The size of the gene in an effective MLST scheme for PCR is very important. In the ‘90s, 400–600 bp was considered the right length for single-run gel-based sequencing [27]. A gene that is too short or too long fails to produce the result [28]. Hence, genes with appropriate sizes were selected that led to the shortlisting of 15 gene candidates.

#### 2.3.7. Unique Alleles of Each Gene

The pragmatic choice of genes for MLST scheme development requires discrimination power. Allele’s profile and sequence types are used to distinguish different strains. A stable gene having more unique alleles can characterize more strains [13]. Alleles for all the selected genes for 502 genomes were labeled using PubMed (https://pubmlst.org/bigsdb?db=pubmlst_S. aureus_seqdef (accessed on 21 October 2022)).

#### 2.3.8. Data Analysis

Sequence alignment was performed using Mega 7 through MUSCLE. The aligned sequences of MLST genes and newly predicted genes were then subjected to different statistical analysis. Statistical analysis was performed using “dnasp5” and “SplitsTree” software. SplitsTree was used to construct a Split decomposition tree, Phi test for recombination, and to calculate the invariant sites (http://www.splitstree.org (accessed on 25 October 2022)) [29]. “Dnasp5” was used to perform the phi test, Tajima’s D test, and Tajima’s dN/dS ratio calculation (http://www.ub.edu/dnasp/index_v5.html (accessed on 25 October 2022)).

##### Significance of Statistical Parameters

The statistical score was used for the selection of candidate genes. The scores are significant in determining the stability of the genes over the course of evolution. The following tests were used to evaluate the genes.

Phi test for Recombination: Phi test is performed to estimate recombination events. Phi test was performed using “Splitstree” in order to estimate the recombination events [29]. The higher rate of recombination is directly related to increased virulence and more pathogenicity [30].

- Tajima’s D value: Tajima’s D value was calculated for already reported MLST scheme and newly predicted genes using “dnasp5”. If the value is zero or closer to zero, it indicates that the observed variation of the population is equal to or closer to the expected. A negative Tajima’s D value shows population expansion. Similarly, a positive value indicates population shrinking or balancing selection. A value between 2 and −2 shows that the genes were driven by neutral selection [31];
- Tajima’s dN/dS ratio: It is a ratio of non-synonymous and synonymous mutations. The ratio is used to distinguish between variants of a population [32];
- dN/dS = (Non-synonymous mutations)/(Synonymous mutations)

The dN/dS ratio was calculated using “dnasp5” software. If the value is greater than 1, it indicates more non-synonymous mutations. However, if the value is smaller than 1, it shows that there are more synonymous mutations.

### 2.4. Primers Construction

The primers for shortlisted genes were constructed using the online primer designing software Primer 3 (http://bioinfo.ut.ee/primer3–0.4.0/ (accessed on 27 October 2022)). The in silico primer’s amplification was also tested using the in silico PCR amplification software (http://insilico.ehu.es/PCR/Amplify.php (accessed on 27 October 2022)).

Primers for new candidate genes were constructed. The following primers were constructed for the suitable candidates shown in Table 1.

### 2.5. Sequence Type Assigning

After the statistical tests, the new gene candidates were finalized. The STs for both the current and new schemes were assigned. For the current scheme, the STs were assigned based on data available on PubMed. For the new scheme, the allelic numbers were assigned manually after the data retrieval of each allele for every gene. The validity of data was confirmed using the online “eburst” tool (http://eburst.mlst.net/ (accessed on 27 October 2022)) to ensure that no two allelic profiles have the same STs [11].

### 2.6. MLST Data Analysis

For analyzing the clonal complexes and studying the phylogenetics of the species the MLST scheme must be presented in a graphical form for analysis. The data analysis can be performed in two ways:

- Based on ST profiles of the genes;
- Based on FASTA sequences of each allele.

The choice of selection of the method depends on the congruency of the alleles. If alleles are congruent then the second method is used, if not, then the other method is used. *S. aureus* is highly clonal, which ultimately refers to the choice of the first method for the analysis. For the very purpose of analysis, “Phyloviz”, which is both downloadable and online software, was used (https://online.phyloviz.net/index (accessed on 28 October 2022)). Two files, the Profile data file and the Accessory data file, were given to the software and the clones of data were analyzed. The Profile data file included the allelic profiles and STs. The Accessory data file included the names of the organisms in the same order as that of the Profile data file. In the Accessory data file, we can add as many details of an organism as we want. This can include the name, origin, source, etc.

## 3. Results

### 3.1. Comparative Pan-Genomic Analysis

#### 3.1.1. *Staphylococcus aureus* Genomic Features and Statistics

As multiple drug and antibiotic resistance has emerged in *S. aureus,* making it a member of the list of major threats to public health by the CDC, and it has become a serious concern for both researchers and the community alike [33]. There has been an exponential increase in the data of *S. aureus* on international databases owing to the advancements and ease in sequencing whole genomes. Currently, 1279 complete genomes and ~31283 incomplete genomes are publicly available. A total of 502/1279 complete genome sequences were downloaded for further analysis as indicated in Table 1 below [34]. The size ranged from 2.66466 Mbp to 3.13248 Mbp with the reference genome, NCTC 8325, having an average size of 2.8 Mbp. The particular bacteria have been evolving and acquiring antibiotic-resistant and virulent genes since the 1940s, with outbreaks occurring mainly in, but not limited to, hospitals and healthcare settings [35].

#### 3.1.2. Pan-Genome Profile Analysis

**Figure 4:**
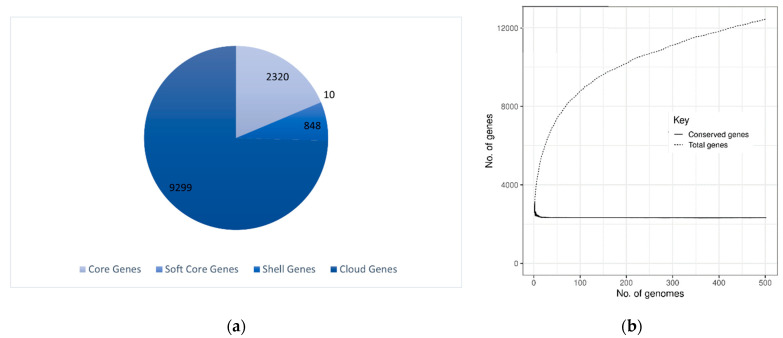
The Pangenome analysis of *S. aureus*: (**a**) Pie chart depicts the core genes, soft core genes, shell genes, and cloud genes. (**b**) The pan vs. core genome plot of 502 strains where the core genome size drops initially and becomes constant afterward; however, the pangenome continues to increase in size.

The pan-genome analysis is a comprehensive study to understand the evolution of bacteria on a molecular and functional basis. The pan-genome of 502 *S. aureus* genomes of a total of 1279 available genomes, is comprised of 12,477 gene families of which 2320 genes (19%) are part of the core genome. According to the COG analysis, which is an appropriate way to identify the functions of individual proteins or protein sets, the majority of the 2320 gene families that make up the core genome are involved in the metabolism transforming nutrients into cellular energy. This represents approximately 19% of the entire genome, the majority of which falls into the category of metabolism by COG analysis. Soft core genes are 10 (0.08%) which represents ~95% incidence of the genome, shell genes are 848 (70%) which cover almost 10–95% of the genome, and 9299 (74%) are numbered as cloud genes which ensure <10% occurrence. The pan and core genome shows fluctuation in the number of pan and core genomes. Overall, the pangenome sizes are not stable and suggest an open pangenome as shown in Figure 4. Pangenome graphs demonstrated a significant expansion in pangenome size upon the addition of new genomes whereas the core genome size drops initially and becomes almost constant and rather stable. Thus, the core genome attains a level of relative stability toward the end of the genomes, making it an ideal component for the prediction of genes to be included in the MLST scheme as well as candidates for vaccine design.

### 3.2. Development of an Improved MLST Scheme

#### 3.2.1. Population Analysis Using Present MLST Scheme

In the population analysis of the current MLST scheme for our 502 genomes, two new *arcC* alleles, which encode for Carbamate kinase, involved in catabolic processes and intermediate metabolism were found for strains FDAARGOS_159 and TCH60. One new *aroE* allele, *aroE* gene encode for Shikimate dehydrogenase, which is again involved in catabolic processes, was found for the NZ15MR0322 strain. One new *gmk* allele, which is essential for the recycling of *gmp,* was identified for the *S. aureus* 2148.N strain. Furthermore, one new allele was found for *tpi* and *yqil* in strains SA40TW and RF122. Consequently, novel alleles and genes were identified during this analysis step, respectively. A total of 10 new STs were found in the available data as manifested in the Additional file Table S2. The numbers of unique alleles of *S. aureus* identified for each of the seven housekeeping genes were *arcC 19*, *aroE* 20, *glpf 18*, *gmk* 15, *pta 19*, *tpi 21*, and *yqil* 23. The mean number of unique alleles is 19.2. The most common allele for different loci was *arcC~3* (77 isolates), *aroE*~3 (74 isolates), *glpf~1* (135 isolates), *gmk*~1 (91 isolates), *pta~4* (79 isolates), *tpi~4* (80 isolates), and *yqil*~3 (78 isolates). The total allelic profiles found in 502 strains are 135. Similarly, the total number of STs found is 52. The most common sequence type found in the data set is ST8 [36]. Furthermore, the dN/dS ratio, which compares synonymous substitution rates (dS) with nonsynonymous substitution rates (dN) to calculate the mode and degree of selection for each allele was also calculated and was demonstrated in Table 2.

##### Limitations of Existing Scheme

The present MLST scheme for *S. aureus* has a few shortcomings according to the literature and our study of the phylogenetics of organisms.

**Figure 5:**
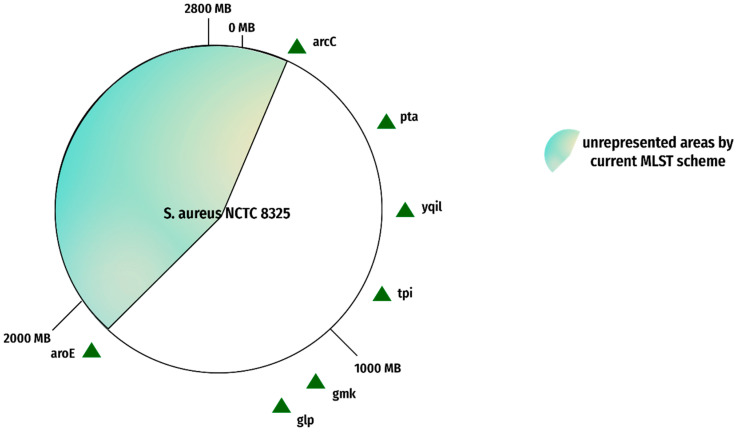
Current MLST gene loci. The figure represents the gene loci of the current MLST scheme on reference genome NCTC 8325. The red portion indicates an unrepresented area, and the green triangles show current MLST gene loci.

**Figure 6:**
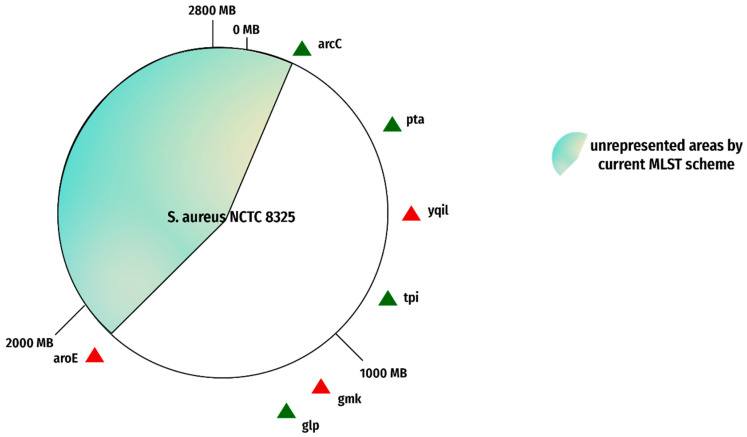
Reported flaws in MLST genes. The figure represents the genes of the current MLST scheme on reference genome NCTC 8325. The red triangles show the genes with reported flaws. The green triangles show the genes with no reported shortcomings.

The present/existing MLST scheme represents a portion of the whole genome, not the complete genome. The complete genome is comprised of many more segments than just those currently represented in the MLST scheme. Approximately 43% of the genomic area goes unrepresented in the present scheme as manifested in Figure 5 and Figure 6. This shortcoming can result in the failure to represent the genome phylogeny [36]. This leaves us with an incomplete picture of how fast evolving or rapidly evolving the species is, which can make it hard for researchers to draw conclusions about its evolutionary history, and it may be impossible to accurately identify all strains from one species.

It is reported that due to 12 bp, overlap in the 3’ region of *gmk* is difficult to amplify in the laboratory which should be conserved, and that led to difficulty in typing of strains. This may be probably due to 12 variations in the 3′ region of 12 bp revealed in our analysis. When the genome was subjected to MLST on PubMed (https://pubmlst.org/bigsdb?db=pubmlst_S. aureus seqdef (accessed on 29 October 2022)), the website did not show any allele number for *yqil*. When it was investigated, the site showed that *yqil* was found truncated from 1–58 in 22 genomes. Furthermore, the statistical analysis revealed that the gene *aroE* and *gmk* of the current scheme have dN/dS values of 12.60328 and 4.06854, respectively. Further details of the statistical analysis of the current scheme are given in Table 3.

#### 3.2.2. Selection of New Genes

**Figure 7:**
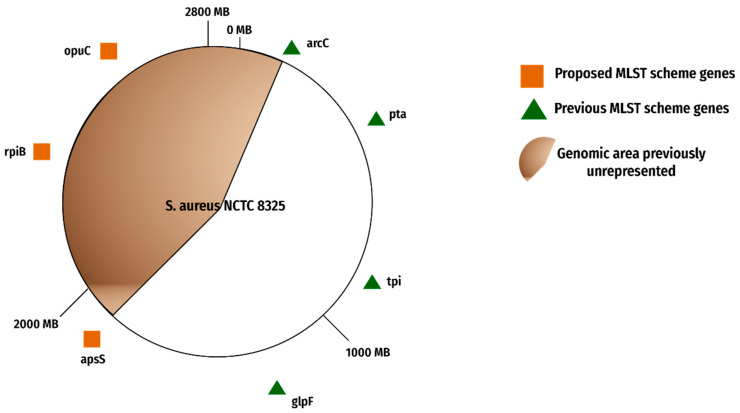
Proposed MLST scheme. The figure shows old, recommended genes and the new proposed gene candidates.

The selected candidate genes denoted the genomic region unrepresented in the previous MLST scheme. Afterward, the genome map was modified in the form of Figure 7.

The initial shortlisting included 130 candidate genes in the unrepresented region, which were further evaluated for their suitability as candidates for the MLST scheme as manifested in Table 4.

These genes were examined for the presence of mobile elements, representing the intra-genome and inter-genomes mobility of the DNA, resulting in a reduction to 55 genes. The genes were further screened for the presence of mobile elements in 100 bp upstream and downstream reducing the number to 23. Later, only nine genes, found in the core genome, were selected after shortlisting based on size and unique alleles.

#### 3.2.3. Statistical Values for New Gene Candidates

For each new candidate, the phi-test for recombination, invariant sites, nucleotide diversity, Tajima’s D, Tajima’s D significance, Tajima’s Non-syn/Syn ratio, Fu and Li’s test, and Fu and Li’s test significance were determined as represented in Table 5.

Based on these statistical scores, only three genes were selected for MLST on the basis of the above-mentioned test parameters.

##### New MLST Scheme

Based on the proposed genes and the current MLST scheme genes that had no reported drawbacks, a new MLST scheme was constructed as shown in Table 6.

This scheme eliminated the genes of the current scheme that included *aroE*, *gmk*, and *yqil*. Three new genes were selected namely, *opuCC*, *aspC*, and *rpiB* on the basis of genome localization and statistical scores.

##### Sequence Type Assigning

The new scheme was assigned STs, and it resulted in 58 STs as manifested in Additional file Table S1. The previous scheme constituted a total of 52 STs, of which 10 were new STs. Hence, the genomic data of 502 sequences showed 42 reported STs and 10 new STs.

#### 3.2.4. MLST Tree Construction

**Figure 8:**
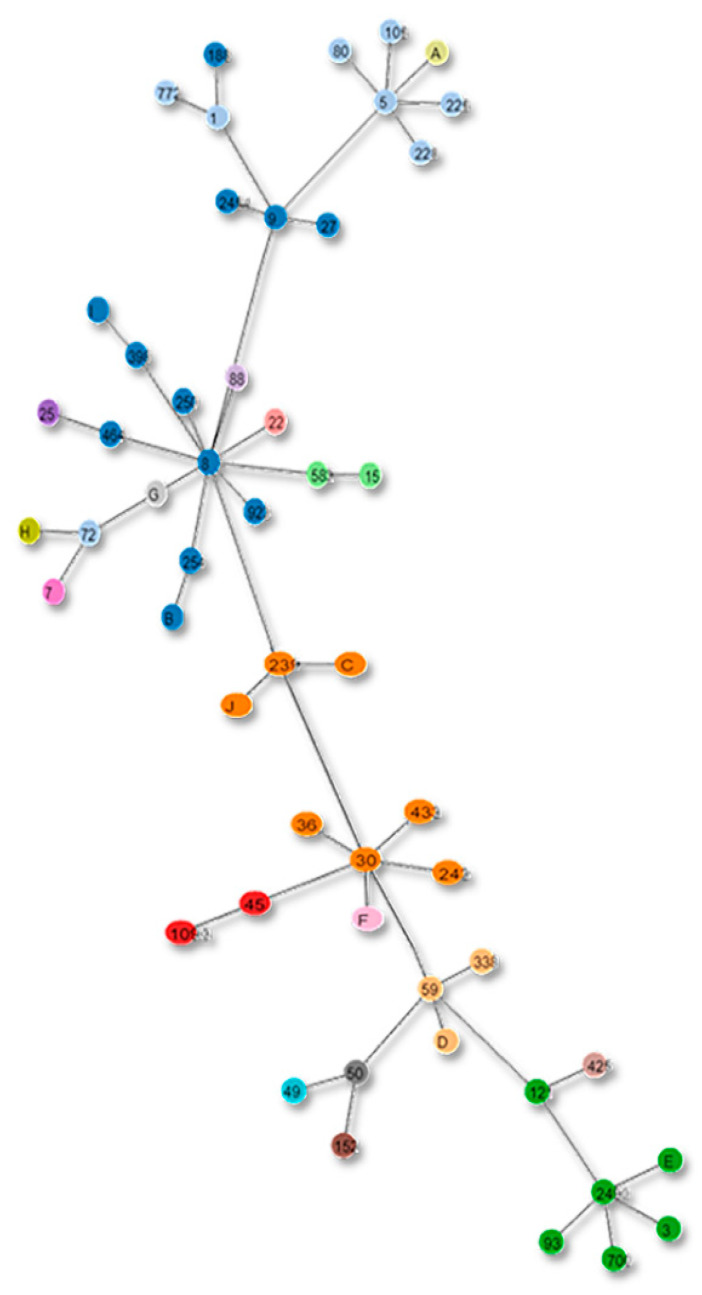
Clonal complexes in the Tree based on the current MLST scheme. The alphabets in the figure show the strains with unreported STs according to the new scheme.

**Figure 9:**
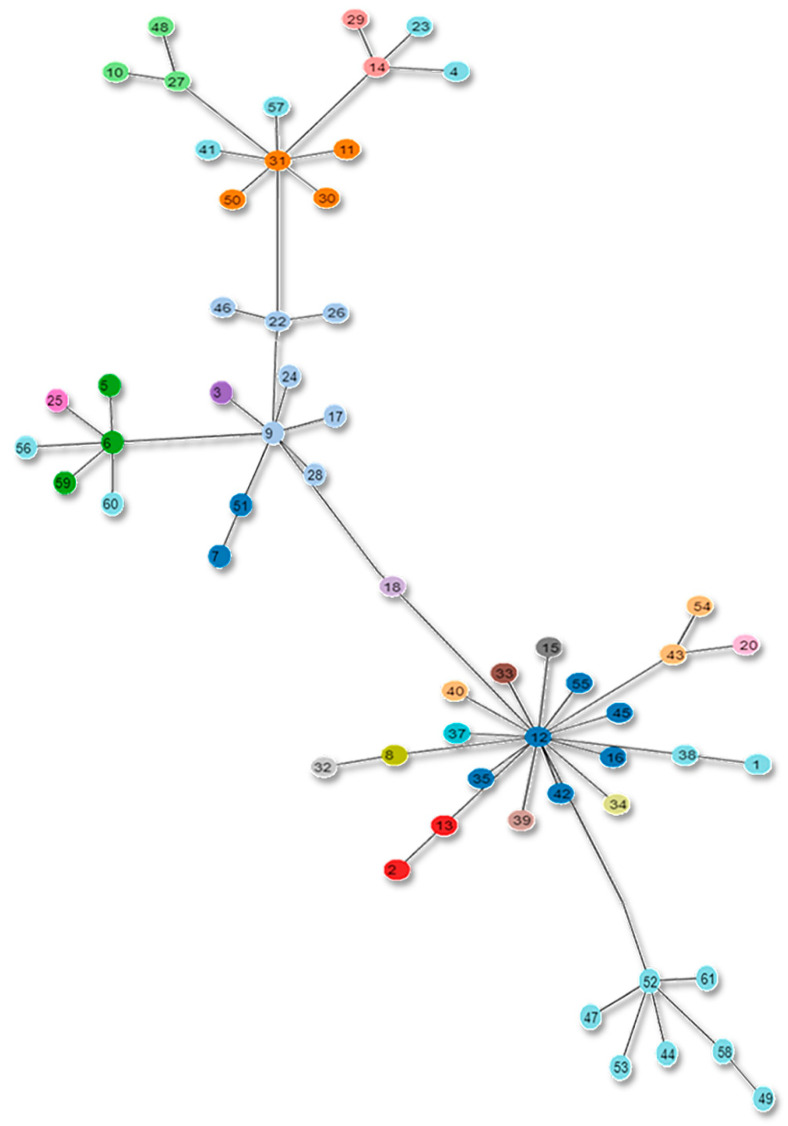
Proposed MLST scheme tree. The figure depicts the new clonal complexes of the proposed MLST scheme in the form of a tree.

MLST trees for current and new schemes were constructed as depicted in Figure 8 and Figure 9.

## 4. Discussion

The advancements in Next Generation Sequencing (NGS) and consequently the increase in the number of genomic data show that there is a need to compile and analyze the data collectively to be able to combat effectively against bacterial pathogens. *S. aureus* is coming into more and more focus as the incidents of the outbreaks have risen over the past decades. Phylogenetic analysis using the sequenced genome data not only helps in determining the evolutionary relationship between the strains but also gives a clue about the way the genome is evolving over time to adapt to its environment. However, the horizontal gene transfer and the acquisition of antibiotic resistance genes make the need of classifying the strains prominent. Several typing methods are currently in use with great room for improvement.

The rapid evolution of bacterial genomes with the passage of time asserts the need for the evaluation of the effectiveness of the current typing methods. Consequently, the MLST schemes also need to be evaluated and revised with time. The revision of schemes makes them suitable for the effective characterization of pathogenic strains. Henceforth, the revision of schemes is a regular process towards the improvement of the typing method. The evaluation and the revision of the MLST schemes improve the resolution power just like the revision of the MLST scheme of the Staphylococcus epidermis gave significant results [36].

According to the literature review and genomic data analysis of *S. aureus*, the current MLST scheme has some shortcomings as well. The polymorphic region of *gmk* and truncated region of *yqil* make it difficult to type the strains in regular laboratory settings. Moreover, the high non-synonymous mutations of the genes indicate less stability of the genes like that of *aroE* and *gmk*. This contradicts the very purpose of MLST genes that are supposed to be stable over the period. Hence, it draws the attention of researchers to look for suitable gene candidates. It is expected that this revision will prove of vital significance and will help us in characterizing the rapidly evolving *S. aureus* genome.

## 5. Conclusions

The current typing methods for bacterial strains have certain limitations and there is no single method available to perform efficiently enough under all situations. Nevertheless, it remains a challenge for the researchers to keep looking at and improving the existing methods until a new or revised method is proposed with ideal typing potentials. The current MLST scheme was evaluated, and an improved scheme was proposed. *gmk*, *yqil*, and *aroE* were excluded from the present scheme, and *opuCC*, *rpiB*, and *aspS* were included in the proposed scheme along with the *arcC, glpF, tpi*, *pta* from the current scheme based on strict exclusion inclusion criteria. According to our research, the proposed genes for the new MLST scheme are relatively stable as shown by the statistical analysis. The Tajima’s D value, Tajima’s non-synonymous to synonymous mutation ratio, and Phi test show promising outcomes for the new scheme. The new scheme also showed more resolution as compared to the previous scheme for our 502-complete genome dataset. Our reported scheme shows 58 different sequence types as opposed to the 52 sequence types that are delineated in the present scheme. The scheme can be used for strain typing in the future.

## Figures and Tables

**Table 1:** Primers for the proposed gene candidates. The table provides the details of the primers and their properties based on which they were selected for the proposed gene candidates.

Sr. No.	Gene	Product Size	Left Primer	Tm	GC	Right Primer	Tm	GC
1	*opuCC*	682	CTGGATGTAGTTT GCCCGGA	59.27	55	TCGCAGC ATAAGGT GGGAAA	59.63	55
2	*aspS*	407	TGTCGTT GCACAAA GTTTAGGT	58.98	45.45	ATCCTAAT GCAATACC GCCATG	58.34	45
3	*rpiB*	419	GATCCAA ACCGCTC AGCAAA	59.12	50	GAACCAC GAACTGT TGGACG	59.42	55

**Table 2:** Unreported alleles and STs. The table represents unreported allelic profiles (STs) in the reported scheme. The orange color highlights the unreported alleles and STs in 502 genomes.

Sr. No.	Strains	*arcC*	*aroE*	*glpf*	*gmk*	*pta*	*tpi*	*yqil*	Unreported STs
1	FDAARGOS_159		4	1	4	12	1	10	
2	FDAARGOS_412	3	32	567	1	4	4	3	
3	M92	2	3	457	1	4	4	3	
4	SA40TW	19	23	15	2	19		15	
5	RF122	6	72	12	43	49	67		
6	TCH60		2	2	2	6	3	2	
7	1971.C01	90	3	1	1	4	4	3	
8	2148.N	484	4	1		4	4	3	
9	NZ15MR0322	3		19	2	20	26	39	
10	V605	2	3	1	238	4	4	3	

**Table 3:** Current MLST scheme. The orange regions in the table indicate genes having unsuitable statistical values.

Sr No.	Gene	Process	Size	Unique Alleles	Phi Test	Tajima’s D	dN/dS Ratio
1	*arcC*	Amino acid transport and metabolism	456	19/502	0.2875	−2.65316	1.19727
2	*aroE*	Coenzyme transport and metabolism	456	20/502	0.2664	−1.17807	12.60328
3	*pta*	Energy production and conversion	474	19/502	0.2723	−1.80001	0.1563
4	*Tpi*	Energy production and conversion	402	24/502	0.2614	−0.40013	−1.47932
5	*glpF*	Carbohydrate transport and metabolism	465	18/502	0.2046	0.37575	−0.69466
6	*yqil*	Inorganic ion transport and metabolism	516	22/502	0.9409	−1.07416	0.97535
7	*gmk*	Carbohydrate transport and metabolism	417	15/502	0.5707	0.57715	4.06854

**Table 4:** Shortlisted gene candidates based on exclusion–inclusion criteria. The table represents the properties of the shortlisted gene candidates that are based on exclusion–inclusion criteria.

Sr. No	Gene Name	Location	Process	Size (bp)	Unique Alleles
1	*ogt*	Cytoplasmic	Replication, Recombination, and Repair	522	26/502
2	*opuCC*	Cell membrane; Lipid-anchor (Probable) [H	Cell wall/membrane biogenesis	942	28/502
3	*ureF*	Cytoplasmic	Post translation Modification, Protein turnover, Chaperones	690	25/502
4	*nrdR*	Cytoplasmic	Transcription	471	18/502
5	*ureE*	Cytoplasmic	Post translation Modification, Protein turnover, Chaperones	453	15/502
6	*rpiM*	NA	50S ribosomal protein L13	438	10/502
7	*rpsE*	NA	30S ribosomal protein S5	501	9/502
8	*rplB*	NA	50S ribosomal protein L2	834	17/502
9	*aspS*	Cytoplasm	Translation, ribosomal structure, and biogenesis	1767	40/502

**Table 5:** Shortlisted gene candidates based on statistically suitable values. The table enlists the nine genes candidates that were shortlisted after determining the statistical values through Phi, Tajima’s, and Fu and Li’s test.

Sr. No	Name	Phi Test	Invariant Sites	Nucleotide Diversity, Pi	Tajima’s D	Significance	dN/dS Ratio
1	*ogt*	×	0.077346	0.02283	0.24068	Not significant, *p* > 0.10	1.03085
2	*opuCC*	✔	0.043663	0.01236	−2.03506	*, *p* < 0.05	0.77636
3	*ureF*	✔	0.021876	0.00683	−2.37067	*p* < 0.01	0.89544
4	*nrdR*	✔	0.006988	0.00341	−1.51133	Not significant, *p* > 0.10	1.34254
5	*ureE*	✔	0.014496	0.00632	−2.33194	**, *p* < 0.01	0.91362
6	*rpiM*	✔	0.003687	0.00191	−1.34752	Not significant, *p* > 0.10	0.66321
7	*rpsE*	✔	8.86E−04	0.00037	−1.93483	*p* < 0.05	N/A
8	*rpiB*	✔	0.002087	0.00106	−2.05422	*p* < 0.05	0.65648
9	*aspS*	✔	0.021738	0.00563	1.62031	0.10 > *p* > 0.05	0.95247

Asterisk * and ** represent significance level: * if *p* ≤ 0.05 and ** if *p* ≤ 0.01.

**Table 6:** Proposed MLST scheme. The table represents the MLST scheme that is based on old and newly proposed candidate genes.

Sr No.	Gene Name	Process	Size	Unique Alleles	Phi Test	Tajima’s D	Non-Syn/Syn Ratio
1	*arc*	Amino acid transport and metabolism	456	19/502	✔	−2.6532	1.19727
2	*Pta*	Energy production and conversion	986	19/502	✔	−1.8	0.1563
3	*Tpi*	Energy production and conversion	368	24/502	✔	−0.4001	−1.4793
4	*glpF*	Carbohydrate transport and metabolism	848	18/502	✔	0.37575	−0.6947
5	*opuCC*	Cell wall/membrane biogenesis	942	28/502	✔	0.04366	−2.0351
6	*aspS*	Translation, ribosomal structure and biogenesis	1767	40/502	✔	−1.6203	0.95247
7	*rpiB*	50S ribosomal protein L2	834	17/502	✔	0.00209	−2.0542

## Data Availability

Not applicable.

## Supplementary Materials

The following supporting information can be downloaded at: https://www.mdpi.com/article/10.3390/genes13112160/s1, Table S1: New STs assigned through Sequence Type Assigning. The table represents the new STs that were assigned based on the new gene alleles. The allele numbers of the previous genes are kept the same to avoid confusion; Table S2: The presence and absence of new genes inserted in a new scheme in the core genomes of *S. aureus* 502 strains.
