# Supplementary material for: Comparative Pan-Genomic Analysis Revealed an Improved Multi-Locus Sequence Typing Scheme for Staphylococcus aureus
Source: Genes (Basel). 2022 Nov 19;13(11):2160. doi: 10.3390/genes13112160 (PMC9690842; doi:10.3390/genes13112160)
Supplement: Supplementary file 1 [file genes-13-02160-s001.zip › Table S1.pdf]

Table S1. New STs assigned through Sequence Type Assigning. The table represents the new STs that were assigned based on the new gene alleles. The allele numbers of the previous genes are kept same to avoid confusion.

Sr No.	Organism name	*arcC*	*glp F*	*pta*	*tpi*	*opuC C*	*asp S*	*rpi B*	ST
1	08-02119	13	1	12	10	21	2	2	1
2	08-02300	5	1	4	6	6	3	1	2
3	08S00974	3	19	20	26	15	8	1	3
4	55-99-44	46	49	13	68	9	5	5	4
5	293G	3	19	20	26	15	8	1	3
6	1625.CO1	3	1	4	4	2	2	8	5
7	2148.C01	3	1	4	4	2	2	2	6
8	ATCC 6538	3	1	29	5	5	3	1	7
9	AUS0325	22	14	12	4	17	1	1	8
10	BB155	46	49	13	68	9	5	5	4
11	C2406	3	1	4	4	1	1	1	9
12	CFSAN007835	10	8	10	3	33	16	11	10
13	CFSAN007847	2	2	6	3	13	7	9	11
14	CFSAN007850	1	1	12	1	5	1	1	12
15	CFSAN007851	1	1	1	1	6	3	1	13
16	CFSAN007883	19	15	19	20	14	7	5	14
17	CFSAN007894	1	1	12	1	5	1	1	12
18	EDCC5458	3	1	4	4	1	1	1	9
19	EDCC5464	7	1	8	8	7	1	10	15
20	FDA209P	3	1	29	5	5	3	1	7
21	FDAARGOS_159	561	1	12	1	5	1	1	16
22	FDAARGOS_412	3	567	4	4	1	1	1	17
23	FORC_012	1	1	4	4	10	1	1	18
24	FORC_026	1	1	1	1	6	3	1	13
25	FORC_027	1	1	12	1	5	1	1	19
26	FORC_039	3	1	1	1	34	4	1	20
27	FORC_040	1	1	4	4	10	1	1	18
28	FORC_045	1	1	1	1	6	3	1	13
29	GD5	3	19	20	26	15	8	1	21
30	GD705	3	19	20	26	15	8	1	21
31	GD1539	3	19	20	26	15	8	1	21
32	GD1677	3	19	20	26	15	8	1	21
33	HC1335	2	1	4	4	1	1	1	22
34	HG001	3	1	4	4	1	1	1	9
35	HZW450	19	15	19	20	31	7	5	23
36	isolate Clinical	3	1	4	4	1	1	1	9
37	Sa_Newman_UoM	3	1	4	4	1	1	1	9

38	ISU926	3	19	20	26	15	8	1	21
39	ISU935	1	1	12	1	5	1	1	19
40	JE2	3	1	4	4	1	1	1	9
41	JH4899	3	1	4	4	1	10	1	24
42	K17	3	1	264	1	3	2	2	25
43	K18	3	1	264	1	3	2	2	25
44	LA-MRSA ST398 E154	3	19	20	26	15	8	1	21
45	M92	2	457	4	4	1	1	1	26
46	MCRF184	10	8	10	3	33	17	11	27
47	MI	1	1	12	1	5	1	1	19
48	NCCP14558	1	1	12	1	5	1	1	19
49	NCCP14562	1	1	12	1	5	1	1	19
50	Newman_D2c	3	1	4	4	1	1	1	9
51	NZAK3	1	1	12	1	5	1	1	19
52	OC8	3	1	4	4	1	12	1	28
53	RIVM6519	1	1	12	1	5	1	1	19
54	SA40TW	19	15	19	540	14	7	5	29
55	Seattle 1945 isolate G477	2	5	6	3	13	7	3	30
56	Seattle 1945 isolate G478	2	5	6	3	13	7	3	30
57	SJTUF_J27	2	2	6	3	13	7	3	31
58	SR434	22	14	12	4	16	2	2	32
59	ST20130938	13	1	12	11	11	1	1	33
60	ST20130939	13	1	12	11	11	1	1	33
61	ST20130940	13	1	12	11	11	1	1	33
62	ST20130941	13	1	12	11	11	1	1	33
63	ST20130942	4	4	5	5	8	1	6	34
64	ST20130943	4	4	5	5	8	1	6	34
65	TMUS2126	1	1	4	4	10	1	1	18
66	TMUS2134	1	1	4	4	10	1	1	18
67	UCI 28	1	1	12	1	5	11	1	35
68	UCI62	1	1	12	1	5	1	1	19
69	USA300_SUR1	3	1	4	4	1	1	1	36
70	USA300-SUR9	3	1	4	4	1	1	1	36
71	USA300-SUR10	3	1	4	4	1	1	1	36
72	USA300-SUR11	3	1	4	4	1	1	1	36
73	USA300-SUR12	3	1	4	4	1	1	1	36
74	USA300-SUR13	3	1	4	4	1	1	1	36
75	USA300-SUR14	3	1	4	4	1	1	1	36
76	USA300-SUR15	3	1	4	4	1	1	1	36
77	USA300-SUR16	3	1	4	4	1	1	1	36

78	USA300-SUR17	3	1	4	4	1	1	1	36
79	USA300-SUR18	3	1	4	4	1	1	1	36
80	USA300-SUR19	3	1	4	4	1	1	1	36
81	USA300-SUR20	3	1	4	4	1	1	1	36
82	USA300-SUR21	3	1	4	4	1	1	1	36
83	USA300-SUR22	3	1	4	4	1	1	1	36
84	USA300-SUR23	3	1	4	4	1	1	1	36
85	USA300-SUR24	3	1	4	4	1	1	1	36
86	USA400-0051	1	1	1	1	6	3	1	13
87	UTSW MRSA 55	3	1	4	4	1	1	1	36
88	ZJ5499	1	1	12	1	5	1	1	19
89	V521	2	1	4	4	1	1	1	22
90	04-02981	1	1	12	25	19	1	1	37
91	08BA02176	3	19	20	26	15	8	1	21
92	25b_MRSA	3	1	4	4	1	1	1	36
93	27b_MRSA	3	1	4	4	1	1	1	36
94	26b_MRSA	3	1	4	4	1	1	1	36
95	29b_MRSA	3	1	4	4	1	1	1	36
96	31b_MRSA	3	1	4	4	1	1	1	36
97	33b	3	1	4	4	1	1	1	36
98	502A	1	1	12	1	18	2	2	38
99	2395 USA500	3	1	4	4	1	1	1	36
100	6850	16	12	13	13	12	1	1	39
101	11819-97	1	1	11	51	4	1	1	40
102	552053	2	2	6	3	23	7	3	41
103	ATCC 25923	2	5	6	3	13	7	3	30
104	aureus ST228 16125	1	1	12	24	5	1	1	42
105	aureus ST228 18583	1	1	12	24	5	1	1	42
106	BA01611	3	1	1	1	4	1	1	43
107	Be62	2	1	4	4	1	1	1	22
108	Bmb9393	2	1	4	4	1	1	1	22
109	CA12	3	1	4	4	1	1	1	36
110	CA15	3	1	4	4	1	1	1	36
111	CA-347	10	8	10	3	33	16	11	10
112	COL	3	1	4	4	1	1	1	36
113	DSM 20231	3	1	4	4	1	1	1	36
114	ECT-R 2	1	1	12	1	5	1	1	19
115	ED98	1	1	12	1	5	1	1	19
116	ED133	6	46	7	50	24	14	12	44
117	FCFHV36	1	1	12		5	1	1	45
118	FORC_001	2	2	6	3	13	7	3	31

119	CN1	1	1	4	4	10	1	1	18
120	GR2	1	1	11	51	4	1	1	40
121	Gv51	2	1	4	4	1	1	1	22
122	Gv69	2	1	4	4	1	2	1	46
123	Gv88	2	1	4	4	1	1	1	22
124	HC1340	2	1	4	4	1	1	1	22
125	H-EMRSA-15	7	1	8	8	7	1	10	15
126	HO 5096 0412	7	1	8	8	7	1	10	15
127	HOU1444-VR	1	1	12	1	5	1	1	19
128	HUV05	3	1	4	4	1	1	1	36
129	ILRI_Eymole11	2	2	6	3	13	7	3	31
130	JH1	1	1	12	1	5	1	1	19
131	JH9	1	1	12	1	5	1	1	19
132	JKD6008	2	1	4	4	1	1	1	22
133	JKD6159	6	44	43	55	27	13	7	47
134	JS395	10	34	26	32	33	18	11	48
135	LGA251	18	6	7	50	32	6	7	49
136	M013	19	15	19	20	14	7	5	14
137	M121	3	1	4	4	1	1	1	36
138	MRSA252	2	2	3	3	13	7	3	50
139	MS4	19	15	19	20	14	7	5	14
140	MSSA476	1	1	1	1	6	3	1	13
141	Mu3	1	1	12	1	5	1	1	19
142	Mu50	1	1	12	1	5	1	1	19
143	MW2	1	1	1	1	6	3	1	13
144	N315	1	1	12	1	5	1	1	19
145	NCTC 8325	3	1	4	4	1	1	1	36
146	NCTC13435	1	1	11	51	4	1	1	40
147	NRS 100	3	1	4	4	5	19	1	51
148	O46 chromosome	6	45	7	14	30	20	1	52
149	RF122	6	12	49	67	28	15	13	53
150	RIVM1295	3	19	20	26	15	8	1	21
151	RIVM1607	3	19	20	26	15	8	1	21
152	RIVM3897	3	19	20	26	15	8	1	21
153	RKI4	3	1	1	22	4	1	1	54
154	SA40	19	15	19	20	14	7	5	14
155	SA268	19	15	19	20	14	7	5	14
156	SA564	1	1	12	1	5	1	1	19
157	SA957	19	15	19	20	14	7	5	14
158	ST228 isolate 10388	1	1	12	24	5	1	1	42
159	ST228 isolate 10497	1	1	12	24	5	1	1	42

160	ST228 isolate 15532	1	1	12	24	5	1	1	42
161	ST228 isolate 16035	1	1	12	24	5	1	1	42
162	ST228 isolate 18341	1	1	12	24	5	1	1	42
163	ST228 isolate 18412	1	1	12	24	5	1	1	42
164	ST398	3	19	20	26	15	8	1	21
165	strain DAR4145	1	1	22	1	5	1	1	55
166	str. Newman	3	1	4	4	1	1	1	36
167	T0131	2	1	4	4	1	1	1	22
168	Tager 104	14	11	13	12	25	9	2	56
169	TCH60	551	2	6	3	22	9	3	57
170	TW20	2	1	4	4	1	1	1	22
171	UA-S391_USA300	3	1	4	4	1	1	1	36
172	USA300_2014.C01	3	1	4	4	1	1	1	36
173	USA300_FPR3757	3	1	4	4	1	1	1	36
174	USA300_TCH1516	3	1	4	4	1	1	1	36
175	USA300-ISMMS1	3	1	4	4	1	1	1	36
176	V2200	3	1	4	4	1	1	1	36
177	VC40	3	1	4	4	1	1	1	36
178	XQ	6	6	7	14	26	6	1	58
179	Z172	2	1	4	4	1	1	1	22
180	1969.N	3	1	4	4	2	2	2	6
181	1971.C01	90	1	4	4	2	2	2	59
182	2148.N	484	1	4	4	20	2	2	60
183	NZ15MR0322	3	19	20	26	15	8	1	21
184	O11	6	45	7	95	29	21	1	61
185	USA300_2014.C02	3	1	4	4	2	2	2	6
186	V605	2	1	4	4	1	1	1	22
187	XN108	2	1	4	4	1	1	1	22
